# Sténose trachéale révélant un lupus érythémateux systémique

**DOI:** 10.11604/pamj.2018.31.139.11651

**Published:** 2018-10-25

**Authors:** Safae Elidrissi, Hind Serhane, Salma Aitbatahar, Hafsa Sajiai, Lamyae Amro

**Affiliations:** 1 Service de Pneumologie, Labo PCIM, UCA, Marrakech, Maroc

**Keywords:** LES, sténose trachéale, anti-DNA natifs, SLE, tracheal stenosis, anti-double stranded DNA (Anti-dsDNA) antibodies

## Abstract

L'atteinte respiratoire dans le cadre du lupus érythémateux systémique (LES) est moins connue que les atteintes cutanées, articulaires et rénales. Le tableau clinique est assez variable et dominé par les atteintes pleurales. La sténose trachéale est inhabituelle voire exceptionnelle au cours de cette pathologie. Nous rapportons un cas assez particulier d'un lupus érythémateux systémique révélé par une sténose trachéale avec un syndrome sec oculo-buccal.

## Abstract (en)

Systemic Lupus Erythematosus (SLE) with respiratory involvement is less well known than cutaneous, articular and renal involvement. Clinical course is quite variable and dominated by pleural involvements. Tracheal stenosis is unusual, if not exceptional in patients with this disease. We here report a quite particular case of systemic lupus erythematosus revealed by tracheal stenosis with dry eye-buccal syndrome.

## Introduction

L'atteinte respiratoire dans le cadre du lupus érythémateux systémique (LES) est moins connue que les atteintes cutanées, articulaires et rénales. L'incidence varie entre 50 et 70% [1]. Tous les éléments anatomiques de l'appareil respiratoire peuvent être affectés, y compris les voies aériennes supérieures et inférieures. La pleurésie est la manifestation respiratoire la plus fréquemment retrouvée au cours du LES. La sténose trachéale ou des bronches souches est rare [2-4]. Nous rapportons un cas exceptionnel de sténose trachéale révélant un lupus érythémateux systémique.

## Patient et observation

**Figure 1:**
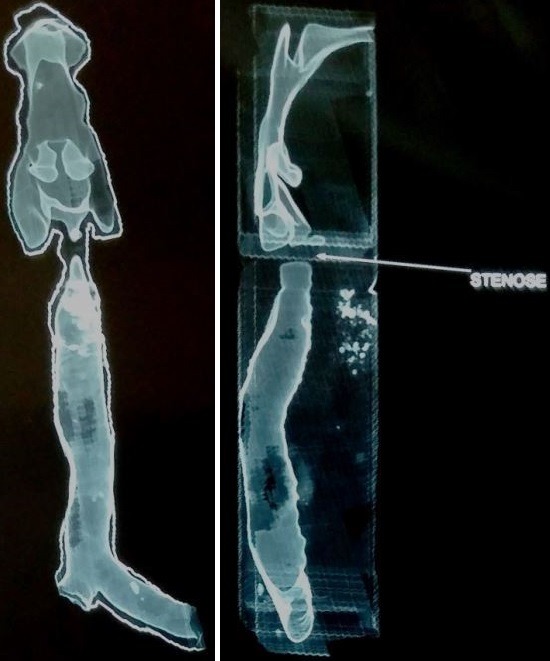
Sténose trachéale sur coupe scannographique avec reconstruction x 3D

**Figure 2:**
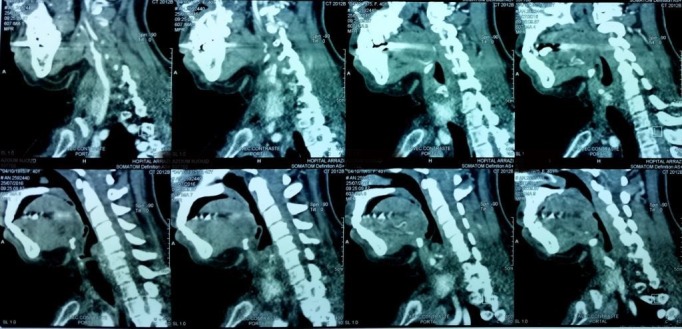
Coupes scannographique sagittales objectivant la sténose trachéale au niveau de l'étage glottique

**Figure 3:**
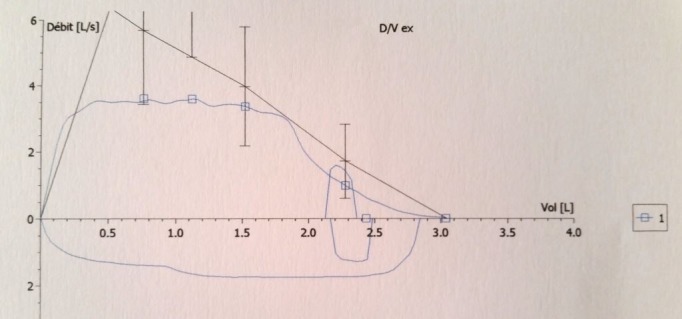
Aspect en plateau de la courbe débit-volume (pléthysmographie)

Il s'agit de Mme. A.N, âgée de 40 ans, suivie depuis 4 ans pour un syndrome sec oculo-buccal sous larmes artificielles, ayant un antécédent de cholécystectomie il y'a 6 ans, admise au service de pneumologie du CHU Mohamed VI de Marrakech pour une dyspnée inspiratoire avec une toux sèche parfois productive ramenant des expectorations muqueuses parfois striées de sang, associées à une dysphonie intermittente et des épistaxis à répétition évoluant dans un contexte de conservation de l'état général, l'examen clinique était normal. La radiographie thoracique a montré un aspect effilé de la trachée. La TDM cervico-thoracique HR avec reconstruction a montré, au niveau de l'étage cervical, une sténose serrée et localisée en diaphragme de la région glottique étendue sur 5mm et qui reste à une distance de 11cm de la carène, et à l'étage thoracique, un foyer de DDB lobaire inférieur gauche avec des nodules et micronodules pulmonaires non spécifiques (Figure 1 et Figure 2). Le bilan biologique a trouvé un syndrome inflammatoire (CRP à 13,34 mg/l et une VS à 50 mm), associé à une anémie hypochrome microcytaire (hémoglobine à 11,2 g/dl), l'urée était à 0,21 g/l, la créatinine à 9,8 mg/l, la calcémie à 94 mg/l, la phosphorémie à 37 mg/l, la calciurie de 24h à 57mg/24h, la phosphaturie de 24h à 732 mg/24h, la protéinurie de 24h était négative à 0,3 g/24h. L'ECBU a trouvé une leucocyturie avec hématurie microscopique et culture stérile. La bacilloscopie était négative. La naso-fibroscopie a objectivé un léger œdème aryténoïdien et rétro-aryténoïdien. La bronchoscopie a trouvé un épaississement des cordes vocales avec une sténose quasi-complète du 1/3 supérieur de la trachée. La biopsie de la sténose trachéale était en faveur d'une inflammation chronique non spécifique. La biopsie des glandes salivaires était sans anomalie. L'examen ophtalmologique a mis en évidence un syndrome sec oculaire modéré. La pléthysmographie a objectivé une distension thoracique avec un aspect en plateau de la courbe débit-volume (VEMS à 85%, VR à 235%, CPT à 135%) (Figure 3). L'Echographie cardiaque n'a pas révélé d'anomalies. Au bilan immunologique, les anti-DNA natifs étaient positifs à un titre de 69,53UI/ml, tandis que les AAN, les anti-MBG, les anti-SSA et anti-SSB, les anti-CCP étaient absents et le facteur rhumatoïde était négatif. A l'immunofluorescence indirecte (IFI), les pANCA à spécificité anti-MPO (anti-myélopéroxydase) étaient positifs (titre estimé à 30 UI/ml). Le diagnostic retenu était le lupus érythémateux systémique. Un traitement à base de corticothérapie orale à raison de 1mg/kg/j de prednisolone est initialement démarré avec dégression progressive, associé à un traitement adjuvant.

## Discussion

La prévalence de l'atteinte respiratoire au cours du LES est de l'ordre de 20 à 90% [5, 6]. La pleurésie est la manifestation respiratoire la plus fréquemment retrouvée au cours du LES touchant 45 à 60% des patients et faisant partie des critères diagnostiques selon la classification de l'American College of Rheumatology (ACR) [1, 7]. Les voies aériennes supérieures et inférieures peuvent être affectées avec des tableaux cliniques variables. La prévalence de l'atteinte des voies aériennes supérieures est retrouvée chez 0,3 à 30 % des malades selon les séries [8-10]. On retrouve des épiglottites aiguës, des laryngites, des œdèmes des cordes vocales, des trachéites nécrosantes, des sténoses précoces post-intubation et des arthrites crico-aryténoïdes. Par contre, la sténose trachéale ou des bronches souches au cours du LES est rarement décrite dans la littérature [2-4] et les données épidémiologiques restent insuffisantes. Le seul cas de sténose trachéale retrouvé dans la littérature est celui rapporté par Zerai *et al.* [11]: il s'agit d'une patiente de 45 ans, suivie depuis 18 ans pour un LES associé à un syndrome de Gougerot Sjögren, qui a présenté 5 mois après une intubation suite à une hémorragie cérébrale du lobe pariétale droit et une pneumonie avec détresse respiratoire, un stridor qui a révélé chez lui une sténose trachéale située à 9,5cm au-dessous des cordes vocales à la TDM avec reconstruction. L'examen histologique de la portion de la trachée reséquée a révélé une inflammation avec fibrose compatible avec un dégât post-intubation. Notre patiente est âgée de 40 ans, suivie depuis 4 ans pour un syndrome sec oculo-buccal, ayant un antécédent de cholécystectomie il y'a 6 ans, la dyspnée inspiratoire était le motif principal d'hospitalisation, l'état général était conservé et l'examen physique était normal. Une sténose trachéale étendue sur 5mm a été fortuitement découverte à la TDM cervico-thoracique HR. La bronchoscopie a trouvé un épaississement des cordes vocales avec une sténose quasi-complète du 1/3 supérieur de la trachée. L'examen histologique de la biopsie de la sténose trachéale était en faveur d'une inflammation chronique non spécifique. Une cause maligne a été écartée. Un bilan immunologique à la recherche d'une atteinte systémique a été réalisé objectivant des anti-DNA natifs fortement positifs (titre de 69,53UI/ml) avec des pANCA à spécificité anti-MPO également positifs (30UI/ml). La sténose trachéale dans notre cas est probablement secondaire à un mécanisme auto-immun au cours de l'évolution du lupus. Par ailleurs, une sténose trachéale post-intubation ne peut être éliminée, mais l'installation différée de la symptomatologie post-intubation en fait une hypothèse diagnostique moins probable.

## Conclusion

La sténose trachéale est rare au cours de l'évolution du LED. Ce mode de révélation et d'autant plus exceptionnel, ce qui fait tout l'intérêt de cette observation.

## Conflits d'intérêts

Les auteurs ne déclarent aucun conflit d'intérêts.

## References

[1] Haupt HM, Moore GW, Hutchins GM (1981). The lung in systemic lupus erythematosus - Analysis of the pathologic changes in 120 patients. Am J Med.

[2] McKnight KM, Adair NE, Agudelo CA (1991). Successful use of tetracycline pleurodesis to treat massive pleural effusion secondary to systemic lupus erythematosus. Arthritis Rheum.

[3] Wohlgelernter D, Loke J, Matthay RA, Siegel NJ (1978). Systemic and discoid lupus erythematosus: analysis of pulmonary function. Yale J Biol Med.

[4] Andonopoulos AP, Constantopoulos SH, Galanopoulou V, Drosos AA, Acritidis NC, Moutsopoulos HM (1988). Pulmonary function of nonsmoking patients with systemic lupus erythematosus. Chest.

[5] D'Cruz D, Khamashta M, Hughes G, Wallace  DJ, Hahn BHH (2002). Pulmonary manifestations of systemic lupus erythematosus. Dubois' Lupus Erythematosus.

[6] Hunninghake GW, Fauci AS (1979). Pulmonary involvement in the collagen vascular diseases. Am Rev Respir Dis.

[7] Keane MP, Lynch JP (2000). Pleuropulmonary manifestations of systemic lupus erythematosus. Thorax.

[8] Nossent JC, Berend K (1998). Cricoarytenoiditis in systemic lupus erythematosus. Scand J Rheumatol.

[9] Langford CA, Sneller MC, Hoffman GS (1997). Methotrexate use in systemic vasculitis. Rheum Dis Clin North Am.

[10] Teitel AD, Mackenzie CR, Stern R, Paget SA (1992). Laryngeal involvement in systemic lupus erythematosus. Semin Arthritis Rheum.

[11] Zerai A, Dosios T, Moutsopoulos HM (1999). Systemic lupus erythematosus and tracheal stenosis. Clin Exp Rheumatol.

